# Clinical Approach to Patients with COVID-19 and Unrecognized Obstructive Sleep Apnea

**DOI:** 10.3390/clinpract14020050

**Published:** 2024-04-18

**Authors:** Melany Ćurić, Frano Marinelli, Vuk Prica, Marijana Pavlović, Igor Barković

**Affiliations:** Department of Pulmonology, Clinic for Internal Medicine, Clinical Hospital Center Rijeka, Tome Strižića 3, 51000 Rijeka, Croatia

**Keywords:** continuous positive airway pressure, COVID-19, obstructive sleep apnea, SARS-CoV-2

## Abstract

**Purpose:** We conducted a retrospective case series of seven male COVID-19 patients with respiratory failure and suspected OSA based on clinical features to evaluate the effects of undiagnosed obstructive sleep apnea (OSA) on COVID-19 outcomes and the response to a continuous positive airway pressure (CPAP) treatment. Cardiorespiratory polygraphy (CRP) and a continuous positive airway pressure treatment were used for diagnosis and management. They confirmed severe obstructive sleep apnea in all patients (apnea/hypopnea index > 30) and improved overnight oxygenation and symptoms at the 1-month follow-up. **Conclusions:** Undiagnosed obstructive sleep apnea may negatively impact COVID-19 outcomes by exacerbating respiratory failure. Recognition and treatment with continuous positive airway pressure can optimize the management of such patients.

## 1. Introduction

Coronavirus disease 2019 (COVID-19) is caused by severe acute respiratory syndrome coronavirus 2 (SARS-CoV-2) [1]. It was first identified in December 2019 in Wuhan, China. It quickly spread globally, leading to a pandemic. The virus primarily spreads through respiratory droplets when an infected person coughs, sneezes or talks, and it can also spread by touching surfaces contaminated with the virus. The virus enters the body through the respiratory tract, primarily infecting cells lining the airways and lungs. It binds to angiotensin-converting enzyme-2 (ACE2) receptors, leading to viral replication and the release of pro-inflammatory cytokines. This triggers an immune response that can cause inflammation and damage to lung tissue, leading to symptoms ranging from mild respiratory illness that includes a dry cough, weakness, fatigue, fever, throat ache, diarrhea, nausea and vomiting, loss of taste and smell to severe pneumonia, acute respiratory distress syndrome (ARDS) and multiorgan failure (MOF) which often leads to a fatal outcome. Additionally, COVID-19 can lead to hypercoagulability and thrombotic complications [1]. As a consequence of bilateral interstitial pneumonia, patients usually present with hypoxemic respiratory failure. In patients with preserved respiratory function, conventional oxygen therapy through a nasal cannula or a mask is sufficient for their recovery. For patients with a severe clinical presentation, a high-flow nasal cannula (HFNC) oxygen therapy or continuous positive airway pressure (CPAP) therapy and non-invasive mechanical ventilation (NIV) may be necessary. In some severely affected patients, endotracheal intubation and invasive mechanical ventilation as well as extracorporeal membrane oxygenation (ECMO) are needed as a life-saving measure [2].

Several comorbidities, such as arterial hypertension, diabetes, cardiovascular diseases, obesity and chronic lung diseases, have been proven to increase the risk of a more negative outcome [3]. One of the comorbidities that also affects the clinical presentation of COVID-19 and its outcome is obstructive sleep apnea (OSA) [4].

OSA is the most common sleep-related breathing disorder [5]. It is characterized with periods of apnea (complete cessation of breathing for at least 10 s) or hypopnea (reduction of airflow for at least 30% lasting more than 10 s) associated with a drop in peripheral oxygen saturation (SpO_2_) of ≥3% (AASM criteria) or ≥4% (CMS criteria) [6]. The pathogenesis of OSA involves various factors, including anatomical abnormalities, such as excess soft tissue in the throat, enlarged tonsils or a narrow airway, which predispose individuals to airway collapse during sleep. When the upper airway collapses, airflow is restricted, leading to oxygen desaturation in the blood. As a result, the body’s oxygen level drops, triggering physiological responses to restore oxygenation, such as awakening, increased respiratory effort and changes in heart rate and blood pressure. These recurrent episodes of hypoxia and awakening disrupt normal sleep patterns and can lead to fragmented sleep, daytime sleepiness and other symptoms associated with OSA. Chronic intermittent hypoxia has been implicated in various adverse health effects, including cardiovascular complications, cognitive impairment, metabolic dysregulation and systemic inflammation. Most symptoms occur during the night and include snoring, restlessness, the cessation of breathing and gasping for air during sleep [5]. During the day, the patients present with daytime sleepiness. It is crucial to emphasize that OSA remains undiagnosed in 75–80% of cases, as individuals often do not pay attention to symptoms, and the condition frequently goes unrecognized as a disease [7]. The prevalence of OSA has been estimated to be present in 14% of men and 5% of women of the population [8]. Together with a clinical history and clinical presentation, the standard method for the confirmation of OSA is overnight polysomnography or cardiorespiratory polygraphy (CRP), which were performed in our study [5]. The apnea/hypopnea index (AHI) per hour is used as the main parameter for determining the severity of the illness [5]. Severity can be divided into mild, moderate and severe sleep apnea. The most effective conservative way of treatment of moderate and severe OSA is with a continuous positive airway pressure (CPAP) device. It is a highly effective treatment that involves wearing a mask connected to a machine that delivers a continuous stream of air pressure to keep the upper airway open during sleep, which allows gas exchange on the alveolo-capillary membrane to continue. This prevents the collapse of throat tissues and maintains uninterrupted breathing throughout the night. The goal of CPAP is to provide a base pressure to overcome static collapsing forces (dependent on the position, skeletal structure, shape and tone of airway tissue), while compensating for variable collapsing forces (dependent on respiratory efforts and flow effects) [9]. The application of CPAP can also decrease atelectasis, increase the surface area of the alveoli, improve ventilation/perfusion matching and oxygenation, as well as ensuring the maintenance of functional residual capacity [10]. Therapy improves oxygenation levels, reduces the frequency of apnea and hypopnea events and promotes better sleep quality. Also, it decreases the risk of cardiovascular complications and improves overall health outcomes in individuals with OSA [5].

Obstructive sleep apnea exacerbates respiratory illnesses such as pneumonia. Compromised respiratory function can lead to a decreased lung capacity, impaired mucociliary clearance and weakened immune response, making individuals more susceptible to respiratory infections like pneumonia. The intermittent hypoxia and oxidative stress associated with OSA can further exacerbate inflammation and tissue damage in the lungs, worsening the severity and outcomes of respiratory illnesses [11,12]. It has also been proven that unrecognized OSA adversely affects patients with COVID-19 pneumonia. Individuals with unrecognized and untreated OSA are at a higher risk of developing severe COVID-19 symptoms, experiencing worse outcomes, such as respiratory failure or mortality, or having a poorer response to COVID-19 treatments compared to individuals without OSA or those receiving treatment for OSA [3,4,13]. Recognizing OSA in patients with respiratory illnesses like COVID-19 pneumonia is essential for optimal management and improved clinical outcomes.

Clinical features, encompassing factors such as anatomy, symptoms, comorbidities, polysomnographic findings and treatment response, play a crucial role in defining the phenotype and severity of obstructive sleep apnea (OSA). These features provide valuable insights into the underlying pathophysiology, prognosis and optimal management strategies for individuals with OSA [14].

The aim of this study was to show the importance of the early detection of symptoms in patients with unrecognized obstructive sleep apnea, the existence of which is of clinical importance in the treatment of patients with respiratory insufficiency, in this case concerning patients with severe bilateral COVID-19 pneumonia.

## 2. Methods

### 2.1. Study Design

The study was designed as a retrospective case series. It included seven male patients who had COVID-19 pneumonia and symptoms indicative of unrecognized obstructive sleep apnea. The study was conducted from 1 September 2020 to 1 September 2021.

Inclusion criteria were the male gender, age between 18 and 75 years, non-smokers, a body mass index (BMI) above 30 kg/m^2^, a COVID-19 infection with pneumonia and respiratory failure defined as a peripheral oxygen saturation (SpO_2_) below 90%. Exclusion criteria were a COVID-19 infection without pneumonia, the female gender, a BMI below 30 and patients with mild respiratory insufficiency.

We identified seven patients who had a similar clinical course and response to therapy and grouped them together, and concluded that it was unrecognized sleep apnea, the treatment of which led to the stabilization of severe pneumonia. The scores were tabulated and subjected to a statistical analysis using Statistica (version 12.0). Descriptive statistics of patients’ demographics and clinical results were reported as a number and relative frequencies.

The patients consented to the study and data processing by signing informed consent forms and the study was approved by the ethics committee of the Clinical Hospital Center.

### 2.2. Patients (Symptoms and Treatment)

All patients were initially admitted to hospital for the treatment of pneumonia due to COVID-19 infection. The assessment was based on a detailed medical history, symptoms and comorbidities. The physical status and vital parameters were evaluated. All patients under went an arterial blood gas (ABG) analysis, laboratory tests and chest X-ray and/or computed tomography pulmonary angiography (CTPA) in addition to PCR testing for SARS-CoV-2, which needed to be positive for each patient. This was the general patient course for patients at the hospital.

All seven patients had certain symptoms, which ledus to suspect that it was unrecognized obstructive sleep apnea. When they fell asleep, heavy breathing would start and their saturation would fall below 80%. When they woke up, the complaints would disappear. All patients were afraid to sleep because they thought they would worsen. They would be sleepy during the day and be clinically better if they exercised with physiotherapists compared to other patients with severe pneumonia, whose respiratory function was worsened by fatigue.

Patients were initially treated with oxygen via a nasal cannula or mask at flow rates ranging from 5 L/min to 15 L/min. Despite oxygen therapy, SpO_2_ levels continued to decline along with a worsening respiratory status, prompting a transition to high-flow nasal cannula (HFNC) therapy. The average HFNC settings ranged from a flow rate of at least 45 L/min to a maximum of 60 L/min, with a fraction of inspired oxygen (FiO_2_) ranging between 50% and 85%. When it was realized that the patients might have unrecognized obstructive sleep apnea and their respiratory status continued to deteriorate despite high pulse and blood pressure values, treatment with a ResMed^®^ Astral^™^ 150 ventilator (ResMed Pty Ltd., Oxford, UK) in CPAP mode was initiated. The CPAP therapy involved delivering positive end-expiratory pressure (PEEP) through the oronasal mask with average pressure values ranging from 8 to 12 cmH_2_O and FiO_2_ between 40 and 60% depending on the assessment of an experienced clinician. The patients experienced respiratory difficulties during the night or day when they would fall asleep. Accordingly, and due to the suspicion that the patients had undiagnosed OSA, we treated them with a CPAP machine only during the night, i.e., during sleep. Between the fifth and tenth day of treatment, further deterioration of the respiratory status was observed in all patients, prompting the initiation of CPAP therapy. Additionally, all patients were treated with corticosteroids (dexamethasone) and remdesivir, as per recommendations. Following improvement, treatment continued with oxygen via a nasal cannula and, subsequently, without oxygen, along with CPAP therapy during the night.

From the perspective of a different individual, it was found out that the patients’ partners noticed that they usually snored during the night and were chocking and gasping for air while sleeping, as well as feeling tired and sleepy during the day. Taking this information into account as well as their BMI and symptoms that were worse during the night, OSA was suspected. All patients filled out the Epworth Sleepiness Scale questionnaire.

Upon the stabilization of their condition and after finishing their isolation period, overnight CRP was performed for six patients in our institution with the Philips^®^ Alice NightOne^™^ sleep testing device and for one patient in another institution for whom we only received the diagnosis confirmation. During the recording, patients were connected to a nasal cannula to monitor airflow, belts around the abdomen and chest were used to record respiratory movements and ECG to monitor heart rate frequency. Additionally, a pulse oximetry sensor was placed on the finger to monitor SpO_2_ levels. All patients who had an apnea/hypopnea index of more than 30/h accompanied by a decrease in SpO_2_ of more than 3% met the criteria for severe sleep apnea.

### 2.3. Follow Up

For the follow up we used the Epworth Sleepiness Scale questionnaire, Modified Medical Research Council Dyspnea Scale (mMRC), a memory card from the CPAP device (AHI/h and CPAP usage), control chest X-ray, ABG and pulmonary function tests.

## 3. Results

In our study, we demonstrated cases of seven male patients admitted to the emergency department complaining mainly of dyspnea, a fever, dry cough, headaches and different degrees of exercise intolerance. The mean age of the patients was 52.1 years old, all of them had a BMI over 30 (mean BMI was 36.24 kg/m^2^), which classified them as obese, and two patients were already diagnosed with arterial hypertension and one with hyperlipoproteinemia. None of the patients had been previously diagnosed with OSA or any other airway disease. All were non-smokers.

**Figure 1:**
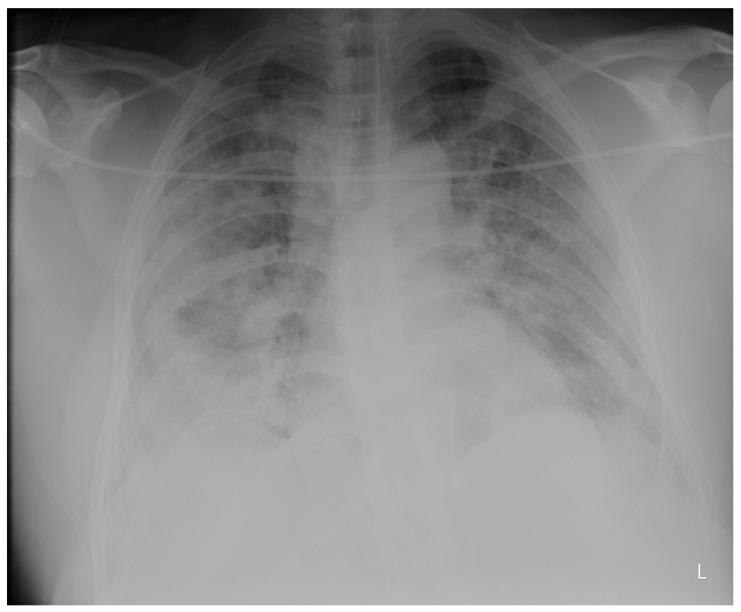
Chest X-ray upon admittance: patient 5. Bilateral inhomogenous infiltrates typical for COVID-19 pneumonia (the “L” orientation marker indicates the left side).

**Figure 2:**
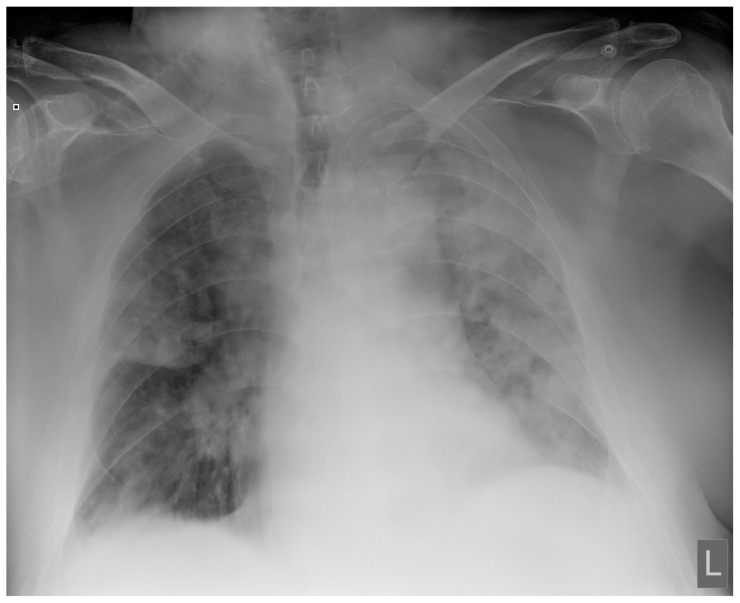
Chest X-ray upon admittance: patient 7. Bilateral inhomogenous infiltrates typical for COVID-19 pneumonia (the “L” orientation marker indicates the left side).

**Figure 3:**
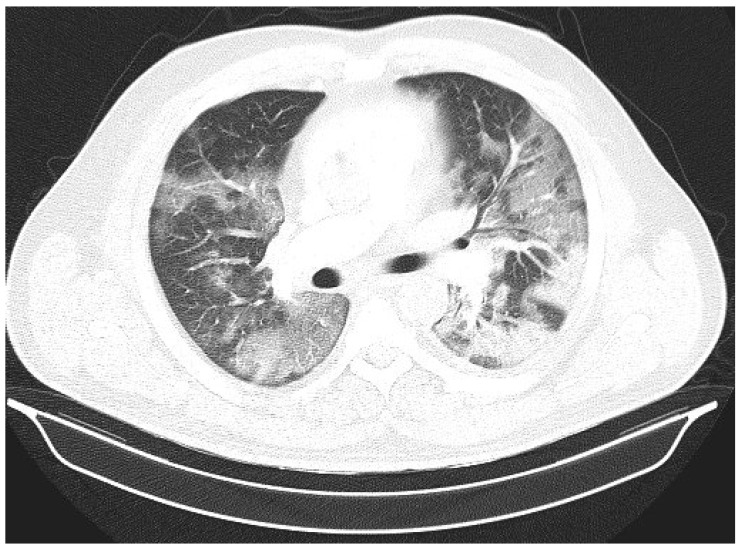
CTPA upon admittance: patient 1. Bilateral ground glass opacifications (severity score: 15–25; chest CT severity score: 14) typical for COVID-19 pneumonia.

**Figure 4:**
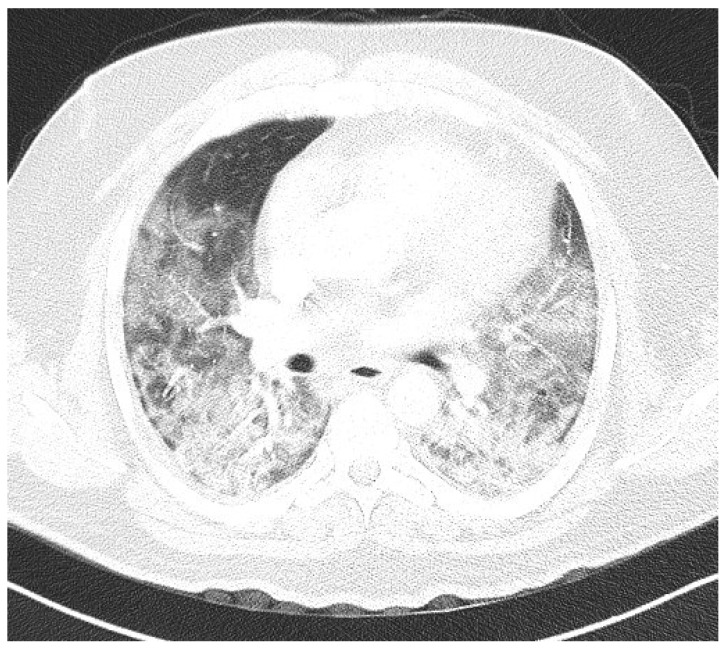
CTPA upon admittance: patient 4. Bilateral ground glass opacifications (severity score: 19–25) typical for COVID-19 pneumonia.

Laboratory findings were mostly normal; C-reactive protein was elevated in all patients with a mean value of 129.7 ± 80.74 mg/L. The arterial blood gas analysis showed hypoxemic respiratory failure. Chest X-rays in all patients showed bilateral inhomogeneous infiltrates (Figure 1 and Figure 2). In three patients, CTPA was performed, which ruled out pulmonary thromboembolism, but ground-glass opacifications with consolidation of the lung parenchyma were described in all of them (Figure 3 and Figure 4) [15].

The patients were initially treated with oxygen via a nasal cannula or mask at flow rates ranging from 5 L/min to 15 L/min. Considering the deterioration of the patient’s condition, treatment was continued with high-flow nasal cannula (HFNC) therapy. The average HFNC settings ranged from a flow rate of at least 45 L/min to a maximum of 60 L/min, with a fraction of inspired oxygen (FiO_2_) ranging between 50% and 85%. Therapy with HFNC improved their symptoms during the day, but their respiratory status remained insufficient during the night, which was mainly seen as a feeling of dyspnea, high respiratory rate and severe drops in SpO_2_ visible on a monitor. Treatment was continued with a ResMed^®^ Astral^™^ 150 ventilator in CPAP mode. The CPAP therapy involved delivering positive end-expiratory pressure (PEEP) through the oronasal mask with average pressure values in our patients ranging from 8 to 12 cmH_2_O and FiO_2_ between 40 and 60%, depending on the assessment of an experienced clinician. Following the administration of CPAP therapy, improvements in the respiratory status were observed, blood pressure and pulse values stabilized and the patients became clinically more stable. Their respiratory rate and SpO_2_ improved with no overnight drops, the patients reported minimal dyspnea and all felt better rested and were better able to perform physical therapy and other essential everyday activities, which ultimately led to their discharge from the hospital with a favorable outcome. With the applied therapy, endotracheal intubation was avoided and further recovery was monitored until clinical resolution. All parameters upon admittance and vital parameters before and after CPAP therapy are shown in Table 1 and Table 2.

Following the stabilization of the patient’s condition, cardiorespiratory polysomnography was performed. Our six patients had an AHI per hour higher than 30, caused by obstructive apneas and hypopneas lasting on average for more than 15 s in all patients, meaning all of them were diagnosed with severe OSA that required immediate treatment. The mean average SpO_2_ was 85.7%. In all patients, SpO_2_ was less than 90% in more than 50% of the time recorded and the minimal SpO_2_ reached below 80% in all of them and in two patients even below 70%. The patients were then titrated on a CPAP device for home use according to the pressures listed in the table. Detailed CRP parameters are shown in Table 3.

**Figure 5:**
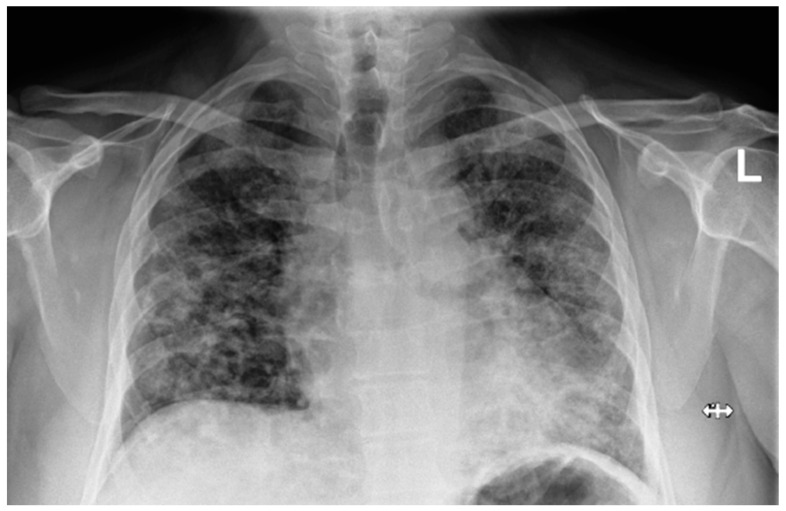
Chest X-ray at the follow up: patient 5. Partial regression of bilateral lung infiltrates (the “L” orientation marker indicates the left side).

After being discharged, at the one-month follow up, the patients were generally feeling much better, were more rested and their daytime sleepiness was reduced; dyspnea on exertion was still troubling several of them. The memory card showed significantly lower AHI per hour in all of them and the device was used over 90% of the time, except for one patient, who used it 68.7% of the time. CPAP therapy was generally well tolerated. Spirometry was normal in five patients and in two patients a restrictive ventilatory defect was noticed, most likely caused by obesity or due to leftover post-COVID infiltrations in the lungs. The ABG analysis was normal in five patients, although in two patients hypoxemic respiratory failure remained. The diffusion capacity for carbon monoxide (DLCO) was reduced (<70%) in four patients and was normal (>70%) in three patients. A chest X-ray described either a partial or complete regression of bilateral lung infiltrates (Figure 5). In two patients, CPAP parameters were slightly adjusted, and in others were left as they were initially prescribed. All follow-up parameters are shown in Table 4.

All patients included in the study had an average age of 52 years and were obese. It is important to note that, upon admission, all patients had acute partial respiratory insufficiency, which was corrected after appropriate treatment. They had bilateral pneumonia described on chest radiography and they were also COVID-19-positive. Initially, all patients were treated with oxygen and then with HFNC, and after symptoms of OSA were recognized, treatment was continued with CPAP therapy, which the patients tolerated extremely well. Symptoms of difficulty breathing were most prominent when patients fell asleep. After using the CPAP machine, symptoms significantly improved. All patients had symptoms suggestive of OSA according to the history provided by their partners. Through diagnostic testing, severe obstructive sleep apnea was confirmed in all patients, warranting treatment with CPAP therapy. On follow-up examinations, patients reported feeling much better with the complete or partial resolution of symptoms and bilateral lung infiltrates and good compliance. A limitation of this study and the presented results was the small sample size and selection bias.

## 4. Discussion

In patients suffering from OSA, there is an increased incidence of developing arterial hypertension (39%), cardiovascular diseases (50%), depression (19%), gastroesophageal reflux disease (18%), diabetes (15%), hypercholesterolemia (10%) and asthma (4%) [8]. Many of these diseases are risk factors for an unfavorable outcome of COVID-19, and it was quickly noticed in intensive care units that the patients who developed a more severe type of the disease and had a worse clinical presentation complicated with the development of acute respiratory insufficiency, which was followed by ARDS in patients who were already suffering from some of the aforementioned comorbidities [3]. One of the comorbidities that can also lead to an adverse outcome of COVID-19 is OSA [4]. This raised a question of how many patients who had unrecognized OSA before suffering from COVID-19 were treated inadequately and, thus, had the potential for an unfavorable outcome.

The retrospective design of the study was limiting due to selection bias, which could have influenced the results. A limitation of our study was the targeted selection of patients with unrecognized OSA without a direct comparison to patients without OSA who had severe COVID-19 pneumonia. Additionally, this was a single-center study. In a prospective study, more attention would be paid to the random selection of patients with previously unrecognized OSA or other respiratory diseases compared to those with previously recognized OSA or patients without OSA. Biomarkers would be specifically determined and the approach to diagnosis and, consequently, the research outcomes would be standardized compared to a retrospective study that was selectively targeted. Future research could include specific biomarkers and diagnostic standards with a multicenter approach.

In our study, all patients presented with severe bilateral pneumonia due to COVID-19 infection, accompanied by consequent acute respiratory failure. We hypothesized that undiagnosed obstructive sleep apnea (OSA) may have had additional repercussions on the severity of the disease itself. After identifying these patients with unrecognized OSA, we initiated appropriate treatment, resulting in improved clinical outcomes.

The impact of untreated OSA on respiratory diseases can be significant and multifaceted. OSA is characterized by recurrent episodes of upper airway obstruction during sleep, leading to intermittent hypoxia, hypercapnia and sleep fragmentation [5,6]. These physiological disturbances can contribute to the development, progression and exacerbation of various respiratory conditions. Untreated OSA can exacerbate pre-existing respiratory diseases such as asthma, chronic obstructive pulmonary disease (COPD) and bronchitis. The recurrent episodes of apnea and hypoxia can trigger bronchoconstriction, airway inflammation and oxidative stress, leading to worsening respiratory symptoms and decreased lung function. Individuals with untreated OSA may be at a higher risk of respiratory infections due to impaired immune function and airway inflammation. The repetitive airway collapse and hypoxia associated with OSA can compromise the mucociliary clearance mechanism, making the airways more susceptible to colonization by pathogens [4,11,16]. Chronic intermittent hypoxia, a hallmark of OSA, can lead to pulmonary vasoconstriction, endothelial dysfunction and the remodeling of the pulmonary vasculature, ultimately resulting in the development of pulmonary hypertension. This condition can further exacerbate respiratory symptoms and increase the risk of cardiovascular complications. Overall, untreated obstructive sleep apnea can have a detrimental impact on respiratory health, exacerbating existing conditions, increasing the risk of respiratory infections and compromising treatment outcomes. The early recognition and appropriate management of OSA are crucial for optimizing respiratory health and improving the overall quality of life. Despite the extensive literature on the overlap of OSA with almost all types of chronic lung diseases (COPD, asthma, chronic cough, interstitial lung disease, pulmonary hypertension and sarcoidosis), there is inadequate recognition and treatment of comorbid sleep-disordered breathing by practitioners encountering chronic lung disease [17,18]. Existing research on the impact of obstructive sleep apnea on respiratory diseases has primarily focused on the known diagnosis of OSA and its effects on a wide range of pulmonary conditions. Similar demographic characteristics of participants were observed in our study; however, there was a difference in diagnostics, since, in most studies, OSA is already a known condition. There exist studies on the impact of unrecognized OSA on postoperative outcomes, indicating worsening respiratory status and cardiovascular incidents among patients with unrecognized OSA in the postoperative period [19].

Given the poorer treatment outcomes for COVID-19 pneumonia in patients with OSA, it is crucial to promptly identify patients with previously unrecognized OSA to initiate timely and appropriate treatment with CPAP therapy compared to other treatment modalities, thereby preventing further disease progression. Since OSA is poorly recognized in the general healthy population, the early recognition of the classic symptoms of this condition by patients, primary care physicians and hospital specialists is necessary to initiate treatment promptly. The aim should be an individualized approach to treatment in patients with specific comorbidities, in our case being obstructive sleep apnea.

## 5. Conclusions

Despite the incomplete understanding of the relationship between OSA and various chronic lung diseases, the treatment of comorbid sleep-related breathing problems remains a promising avenue for improving not only quality of life, but also, potentially, morbidity and mortality in these patients. Further research is needed, involving a larger number of patients in randomized, double-blind, multicenter studies, to demonstrate the impact of unrecognized obstructive sleep apnea on various respiratory diseases, particularly in the case of pneumonia due to COVID-19. In the future, the goal is to standardize diagnostic methods for the early and timely recognition of OSA, develop specific biomarkers and raise awareness in the population about this condition.

## Figures and Tables

**Table 1:** Patient characteristics upon admittance.

Patient	1	2	3	4	5	6	7	Mean ± SD
Sex	Male							
Age (years)	47	51	36	41	50	73	67	52.1 ± 12.38
Comorbidities	Arterial hypertension	N/A	N/A	N/A	N/A	N/A	Arterial hypertension, hyperlipoproteinemia	/
BMI (kg/m^2^)	40	36.4	33.8	45.6	32.5	35.2	30.2	36.24 ± 4.77
C-reactive protein (mg/L)	79.2	86.1	47.2	230.4	156.7	65.3	243	129 ± 74
ABG analysis	Hypoxemic respiratory failure							
Chest X-ray	Bilateral inhomogeneous infiltrates							
CTPA	Bilateral ground glass opacifications (severity score 15/25) *	N/A	N/A	Bilateral ground glass opacifications (severity score 19/25)	N/A	Bilateral ground glass opacifications (severity score 16/25)	N/A	/
Initial HFNC treatment (oxygen flow [L/min], FiO_2_ [%])	60 L/min, 50%	55 L/min, 60%	60 L/min, 80%	60 L/min, 85%	50 L/min, 70%	50 L/min, 50%	45 L/min, 50%	55 L/min ± 5.62, 65 ± 13.81
Initial CPAP treatment [cmH_2_O], FiO_2_ [%]	8 cmH_2_O, 50%	12 cmH_2_O, 40%	8 cmH_2_O, 50%	10 cmH_2_O, 60%	10 cmH_2_O, 55%	8 cmH_2_O, 40%	10 cmH_2_O, 50%	9 cmH_2_O ± 1.39, 50% ± 6.67

* CT severity score of COVID-19 [14].

**Table 2:** Vital parameters before/after CPAP therapy.

Patient	1	2	3	4	5	6	7
Pre CPAP							
SpO_2_/%	75%	80%	83%	80%	88%	90%	89%
Blood pressure/mmHg	150/80	140/70	155/95	185/95	180/90	165/85	180/100
Heart rate/min	110	125	90	140	125	108	110
Respiratory rate/min	25	36	28	40	36	26	28
Post CPAP							
SpO_2_/%	90%	92%	94%	91%	95%	94%	96%
Blood pressure/mmHg	110/80	115/70	125/80	140/90	130/70	125/85	130/85
Heart rate/min	90	85	80	100	90	75	80
Respiratory rate/min	16	18	16	20	16	12	14

**Table 3:** Cardiorespiratory polygraphy results and initial continuous positive airway pressure therapy.

Patient	1	2	3	4	5	6	7	Mean + SD
AHI per hour	36	56	32,6	*	35.6	40.3	78.8	46.5 ± 16.21
Average apnea/hypopnea duration (seconds)	18.5 s	17.9 s	22 s		15.2 s	30.4 s	36 s	23.3 ± 7.57
Average SpO_2_	85%	82%	88%		87%	86%	86%	85 ± 1.88
Percentage of time SpO_2_ was under 90%	65.9%	99.8%	56%		55.4%	98.8%	86.9%	77.2 ± 18
Minimal SpO_2_	73%	63%	78%		79%	75%	64%	72 ± 6.32
CPAP device (minimal and maximal pressure [cmH_2_O])	Min 5 cmH_2_O Max 15 cmH_2_O	Min 5 cmH_2_O Max 12 cmH_2_O	Min 6 cmH_2_O Max 12 cmH_2_O	Min 5 cmH_2_O Max 12 cmH_2_O	Min 6 cmH_2_O Max 11 cmH_2_O	Min 5 cmH_2_O Max 15 cmH_2_O	Min 5 cmH_2_O Max 11 cmH_2_O	Min 5.3± 0.45 Max 12.57 ± 1.5

* CRP conductedin another institution, only diagnosis confirmation received.

**Table 4:** One-month follow-up.

Patient	1	2	3	4	5	6	7	Mean
Spirometry (FVC *)	Normal FVC 85%	Normal FVC 94%	Normal FVC 88%	Restrictive ventilatory defect FVC 65%	Normal FVC 84%	Restrictive ventilatory defect FVC 54%	Normal FVC 90%	FVC 80 ± 13.63%
DLCO	61%	76%	82%	53%	63%	48%	110%	70 ± 19.57%
ABG	Normal	Normal	Normal	Hypoxemic respiratory failure	Normal	Hypoxemic respiratory failure	Normal	/
AHI per hour	2.4	2.8	3.6	4.8	3	4.5	7.2	4.04 ± 1.59
CPAP usage	100%	100%	94%	68.7%	100%	91%	92%	92 ± 10.5%
Chest X-ray infiltrate regression	Partial	Partial	Complete	Partial	Complete	Partial	Complete	/
CPAP parameter change (minimal and maximal pressure [cm H_2_O])	Min 5 cmH_2_O Max 10 cmH_2_O	Min 5 cmH_2_O Max 10 cmH_2_O	N/A	N/A	N/A	N/A	N/A	/
Subjective feeling (questionnaires: Epworth and mMRC)	Better, rested, less daytime sleepiness.	Still feeling tired, less daytime sleepiness.	Feeling rested, still has dyspnea on exertion.	Feeling better, still has dyspnea on exertion.	Feeling better, no daytime sleepiness.	Feeling better, still has dyspnea.	Feeling much better, still lacks strength.	/

* Forced vital capacity.

## Data Availability

The original contributions presented in the study are included in the article, further inquiries can be directed to the corresponding author.

## Abbreviations

ABG	Arterial blood gas
ACE-2	Angiotensin-converting enzyme-2
AHI	Apnea/hypopnea index
ARDS	Acute respiratory distress syndrome
BMI	Body mass index
COVID-19	Coronavirus disease 2019
CPAP	Continuous positive airway pressure
CRP	Cardiorespiratory polygraphy
CTPA	Computed tomography pulmonary angiography
DLCO	Diffuse lung capacity for carbon monoxide
ECMO	Extracorporeal membrane oxygenation
FiO_2_	Fraction of inspired oxygen
HFNC	High-flow nasal cannula
mMRC	Modified Medical Research Council
MOF	Multiorgan failure
NIV	Noninvasive ventilation
OSA	Obstructive sleep apnea
PEEP	Positive end-expiratory pressure
SARS-CoV-2	Severe acute respiratory syndrome coronavirus 2
SpO_2_	Peripheral oxygen saturation
